# Measles susceptibility of marriage migrant women in Korea

**DOI:** 10.4178/epih.e2022031

**Published:** 2022-03-12

**Authors:** Sooyeon Kim, Sun A Kim, Hanbich Hong, Seong Ryeong Choi, Hae-Young Na, Sung Un Shin, Kyung-Hwa Park, Sook In Jung, Min-Ho Shin, Sun-Seog Kweon, Seung Ji Kang

**Affiliations:** 1 Jeonnam Communicable Disease Management Support Team, Muan, Korea; 2 Honam Regional Center for Disease Control and Prevention, Gwangju, Korea; 3 Jeollanam-do Institute of Health and Environment, Muan, Korea; 4 Department of Internal Medicine, Chonnam National University Hospital, Gwangju, Korea; 5 Department of Internal Medicine, Chonnam National University Medical School, Hwasun, Korea; 6 Department of Preventive Medicine, Chonnam National University Medical School, Hwasun, Korea

**Keywords:** Measles, Migration and immigration, Seroepidemiologic studies, Vaccines

## Abstract

International migrants could be considered a risk group susceptible to vaccine-preventable diseases. We conducted a measles seroprevalence study among 419 marriage migrant women living in Sinan-gun and Wando-gun, South Jeolla Province, located in the southwestern part of Korea. The overall seroimmunity was 92.8%. The seroimmunity varied considerably according to the country of origin and increased with age. Our current analysis could be valuable in the context of discussions concerning vaccination policies for immigrants in Korea.

Measles elimination in Korea was confirmed by the World Health Organization in 2014. However, small sporadic outbreaks of measles continue to occur, mostly driven by imported cases [1]. Measles introduced from abroad spreads to susceptible populations, such as infants or young adults who are insufficiently vaccinated or individuals in whom the vaccine effect has waned. The number of foreign-born individuals in Korea has increased sharply (1,271,807 foreigners with long-term stays in 2019), of whom approximately 10% are marriage migrants [2]. Marriage migration has increased rapidly since the mid-1990s as the government officially encouraged international marriages between foreign women and Korean men to resolve the rural bride shortage. Earlier, most foreign brides were *Chosunjok* (ethnic Koreans from China), but recently, their countries of origin have diversified. As of January 2019, out of a total of 132,748 foreign-born women married to Korean men and living in Korea, the most common country of origin was China (34.7%), followed by Vietnam (30.4%), Japan (9.4%), Philippines (8.6%), Cambodia (3.2%), Thailand (3.3%), and others (10.3%) [3]. Measles vaccination rates worldwide have increased over time, but are still lower in low-income or middle-income countries than in developed countries. The estimated first-dose measles-containing vaccine (MCV1) coverage in Vietnam was 84.4% to 93.3% between 2000 and 2019, but administration of the second-dose measlescontaining vaccine (MCV2) only started in 2007 [4,5]. The estimated MCV1 coverage in Cambodia was 63.7% to 91.1% between 2000 and 2019, and MCV2 administration only started in 2012 [4,5]. These suboptimal measles vaccine coverage rates in the countries of origin of marriage migrant women suggest the possible influx of measles-susceptible populations into Korea. Since marriage migrant women frequently become pregnant, give birth, take care of children, and travel overseas to their country of origin, immunity to measles is particularly important in this group. However, no information has yet been published regarding measles immunity in marriage migrant women in Korea. To address this issue, we conducted a seroprevalence study in marriage migrant women from July to October 2019.

Participants were recruited among marriage migrant women registered at multicultural family support centers in Sinan-gun and Wando-gun, which are county-level divisions in South Jeolla Province, located in the southwestern part of Korea. Their serostatus was evaluated by testing serum immunoglobulin G (IgG) levels using chemiluminescence immunoassay performed on LIAISON^®^ XL (DiaSorin, Dietzenbach, Germany) at Seoul Clinical Laboratories. IgG values of > 16.5 AU/mL, 13.5-16.4 AU/mL, and < 13.5 AU/mL were regarded as positive, equivocal, and negative, respectively. We considered positive test results as representing immunity to measles. All participants were offered consecutive immunization, if necessary, based on their IgG status. Demographic data and history of previous measles infection and vaccination were obtained via a questionnaire. Of 596 marriage migrant women registered in multicultural family support centers, 419 (184 from Sinan-gun and 235 from Wando-gun) participated in the study. No participants reported being infected with or vaccinated against measles after arriving in Korea. The median age of the participants was 34 years (range, 19-59; interquartile range, 30-40). More than half of the participants originated from Vietnam (51.1%), followed by China (16.9%), the Philippines (14.1%), Cambodia (11.5%), Thailand (2.6%), and other countries (3.8%) (4 from Japan, 4 from Laos, 3 from Kazakhstan, and one 1 each from Taiwan, Myanmar, Indonesia, Russia, and Kyrgyzstan). Of the total 419 serum samples tested, 92.8% were positive, 1.9% were equivocal, and 5.3% were negative for measles IgG. Seropositivity for measles was significantly lower in those aged < 30 years (Table 1). In addition, immunity to measles varied with the country of origin (p< 0.01). Of the 5 countries from which 5 or more participants originated, the lowest number of individuals with seropositivity was observed among immigrants from Cambodia (79.2%), followed by China (84.5%), Vietnam (96.7%), and the Philippines and Thailand (100% each). All individuals from Laos, Japan, Myanmar, Indonesia, Russia, and Kyrgyzstan tested positive for measles IgG. One of the 3 individuals from Kazakhstan and the 1 individual from Taiwan tested negative for measles IgG. Figure 1 depicts the distribution of immune status according to country of origin and age groups (< 30 vs. ≥ 30 years old).

In this study, we found that the population aged < 30 years, particularly individuals from Cambodia, had significantly lower immunity for measles than the ≥ 30-year-old population. For Cambodia, we found 1 nationwide measles serosurveillance study performed in 2012 [6]. According to this study, seropositivity to measles was 95.9% overall, 89.6% for ages 15-19, and 96.1% for ages 20-24. This finding is different from that of our study, which showed only a 40% seropositive rate among Cambodian marriage migrant women aged 23-29 years. Since relatively few Cambodian migrants were investigated in our study, more extensive surveillance should be conducted to explain this difference. However, several causes can be inferred based on the limited data. Although we did not collect detailed data on regions of origin within countries, area of origin might affect the likelihood of immunity to measles. Regional differences in seropositivity within Cambodia were presented in the study by Mao et al. [6]. Different MCV1 coverage rates between rural and urban areas were also shown in the study by the Local Burden of Disease Vaccine Coverage Collaborators [4]. The estimated MCV1 coverage in Cambodia from 1990 to 2000 was 34-65%, and the number of reported measles cases markedly decreased in the 1990s compared to the 1980s (32,240 cases in 1980 and 2,470 cases in 1990) [5]. Therefore, some extent of measles immunity in people born after 1990 may have been acquired through vaccination and not via infection. Since our study was performed 7 years after the study by Mao et al. [7], waning vaccine-induced immunity may be another reason for the observation of lower measles immunity in young adults in our study. However, in order to obtain a clearer explanation, further research among Cambodian immigrants in Korea is warranted. The most recent study performed in Vietnam showed that seropositivity to measles was 89% in those aged 20-24 years and ≥ 98% in those aged 25-70 years, similar to the finding of our study [7]. For China, although there were differences depending on the survey period and region, the overall reported seropositivity to measles was 80-90%, which is comparable to our results [8]. In Thailand, aligning with our observations among Thai migrants, Wanlapakorn et al. [9] reported a high seroprotection rate (98.7%) for measles in those aged > 30 years.

In young adults, low immunity to measles was observed in this study and other studies conducted in marriage migrants’ countries of origin [6-9]. For Korea, the 2-dose measles-mumps-rubella vaccination rate has been maintained at > 95% since 1996, but immunity to measles in adolescents and young adults was significantly lower (49-70%) than that in older adults [10]. This is presumably related to faster waning of vaccine-induced antibodies compared to antibodies induced by natural infection. Because of these susceptible pockets, sporadic outbreaks of measles continue to occur even in countries with high measles vaccination rates, mostly driven by imported cases [1]. In Southeast Asian countries, suboptimal vaccination coverage, combined with reduced measles circulation, has contributed to the development of a susceptible young population and the resurgence of measles outbreaks. Cambodia received its measles elimination status in 2015, but resurgence was reported between 2019 and 2020 [5]. Intermittent surges were also observed in Vietnam and Philippines in 2014 and 2018, respectively [5]. Therefore, to prevent measles resurgence, continuous monitoring of immunity in communities, including foreign-born populations who may have different serostatus, is essential. Surveillance for immunity in marriage migrant women is particularly important because they are usually young, are more likely to travel to their home countries that have measles epidemics, and are in frequent contact with infants, who are susceptible to measles.

Our study had several limitations. Since this survey was only conducted in 2 counties in the southwestern part of Korea, our cohort was not representative of all marriage migrant women in Korea. According to data released by the Korea Immigration Service in January 2019, a total of 132,748 foreign-born women married to Koreans were registered, but only 419 marriage migrant women were included in this study [3]. Nationally, the most common country of origin was China, followed by Vietnam and Cambodia, but in our study, Vietnam was the most common country of origin. Owing to the small number of subjects from each country, our results could not represent overall measles seroprevalence based on marriage migrants’ country of origin. Since the vaccination and infection rates can vary based on region, epidemiological characteristics, and socioeconomic status, applying seroprevalence data obtained from a small number of subjects to the general population has limitations. However, despite these limitations, our results raise concerns regarding measles immunity and show that measles immunity could be particularly low in certain populations. It is necessary to establish policies to monitor immunity against measles in marriage migrant women and vaccinate them, if necessary.

**Ethics statement**

This study protocol was approved by the Institutional Review Board (IRB) of Chonnam National University Hwasun Hospital (CNUHH 2019-114). Informed consent was confirmed by the IRB.

## Figures and Tables

**Figure 1.**
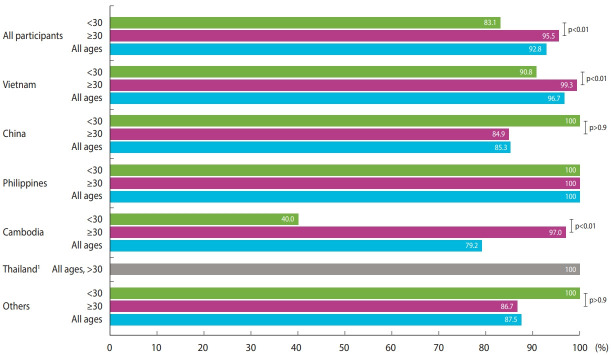
Prevalence of measles immunoglobulin G seropositivity by country of origin and age group. ^1^All participants from Thailand were >30 years old.

**Table 1.** Measles seroprevalence among marriage migrant women according to age group

Age (yr)	n	% of positive results	p-value
Total	419	92.8	0.001
19-29	89	83.1	
30-39	215	95.8	
40-49	95	95.8	
50-60	20	90.0	

## References

[1] Yang TU, Kim JW, Eom HE, Oh HK, Kim ES, Kang HJ (2015). Resurgence of measles in a country of elimination: interim assessment and current control measures in the Republic of Korea in early 2014. Int J Infect Dis.

[2] https://kosis.kr/easyViewStatis/customStatisIndex.do?vwcd=MT_TM1_TITLE&menuId=M_03_01.

[3] https://www.immigration.go.kr/immigration/1569/subview.do.

[4] Local Burden of Disease Vaccine Coverage Collaborators (2021). Mapping routine measles vaccination in low- and middle-income countries. Nature.

[5] https://apps.who.int/immunization_monitoring/globalsummary.

[6] Mao B, Chheng K, Wannemuehler K, Vynnycky E, Buth S, Soeung SC (2015). Immunity to polio, measles and rubella in women of child-bearing age and estimated congenital rubella syndrome incidence, Cambodia, 2012. Epidemiol Infect.

[7] Hachiya M, Do TH, Huynh KM, Vien QM, Hoang TT, Nguyen BT (2020). Population immunity for measles, rubella, mumps, and varicella among adults in Khanh Hoa province, Socialist Republic of Vietnam. Int J Infect Dis.

[8] Zhang Z, Chen M, Wang Y, Li J, Li X, Lu L (2019). Seroepidemiology of measles in Beijing, China: a cross-sectional study. Hum Vaccin Immunother.

[9] Wanlapakorn N, Wasitthankasem R, Vichaiwattana P, Auphimai C, Yoocharoen P, Vongpunsawad S (2019). Antibodies against measles and rubella virus among different age groups in Thailand: a population-based serological survey. PLoS One.

[10] Kang HJ, Han YW, Kim SJ, Kim YJ, Kim AR, Kim JA (2017). An increasing, potentially measles-susceptible population over time after vaccination in Korea. Vaccine.

